# Baseline and changes in serum uric acid independently predict glucose control among community-dwelling women

**DOI:** 10.1186/s13098-018-0356-z

**Published:** 2018-07-13

**Authors:** Ryuichi Kawamoto, Daisuke Ninomiya, Asuka Kikuchi, Taichi Akase, Teru Kumagi

**Affiliations:** 1 Department of Community Medicine, Ehime University Graduate School of Medicine, Toon, 791-0295 Japan; 2 Department of Internal Medicine, Seiyo Municipal Nomura Hospital, 9-53 Nnomura, Nomura-cho, Seiyo, 797-1212 Japan

**Keywords:** Serum uric acid, Glucose control, Hemoglobin A1c, Retrospective cohort study, Women

## Abstract

**Background:**

Elevated serum uric acid (SUA) levels are associated with glucose control. However, whether baseline and changes in SUA predict long-term follow-up glucose control [e.g., glycated hemoglobin (HbA1c)] remains unclear.

**Methods:**

The subjects comprised 393 women aged 71 ± 8 years and 279 men aged 71 ± 10 years from a rural village. We have identified participants who underwent a similar examination 11 years prior, and subjects were divided into four groups based on the tertiles of baseline and changes in SUA, and examined the relationship between baseline and changes in SUA, and glucose control evaluated by follow-up HbA1c after 11-years.

**Results:**

In both genders, follow-up SUA were significantly higher in Group 4 (i.e., women: Group 4, baseline SUA ≥ 4.0 mg/dL and changes in SUA ≥ 0.8 mg/dL; men: Group 4, ≥ 5.3 mg/dL and ≥ 0.4 mg/dL) than in the other Groups, but eGFR was significantly lower. Only in women, there were significant differences among the four groups regarding follow-up HbA1c, and follow-up HbA1c was highest in Group 4. In addition, the interaction between baseline and changes in SUA (F = 5.391, *p *= 0.021) as well as baseline low-density lipoprotein cholesterol (LDL-C) (F = 13.793, *p *< 0.001), estimated glomerular filtration ratio (F = 10.715, *p *= 0.001), HbA1c (F = 118.285, *p *< 0.001), SUA (F = 9.457, *p *= 0.002), and changes in SUA (F = 7.757, *p *= 0.006) was a significant and independent determinant of follow-up HbA1c. Multivariate-adjusted follow-up HbA1c (*p *= 0.002) were significantly higher in Group 4 than the other groups.

**Conclusions:**

These results suggested that combined assessment of baseline and changes in SUA provides increased information for long-term predictive glucose control, independent of other confounding factors in community-dwelling women.

**Electronic supplementary material:**

The online version of this article (10.1186/s13098-018-0356-z) contains supplementary material, which is available to authorized users.

## Background

Uric acid (UA) is the final metabolism product of endogenous purine catabolism in humans that is responsible for the production of UA and damage of free radicals [1]. The enzymes involved in UA production are also responsible for oxidative stress [1] and it was evidenced that serum UA (SUA) might be dependently or independently related to different multifactorial disorders. A recent meta-analysis revealed that baseline SUA was also closely associated with an increased risk for subsequent development of impaired fasting glucose (IFG) [2] and type 2 diabetes [2–5]. While others found no association between high SUA and type 2 diabetes [6, 7]. From the cohort Study over 4 years, Cicero et al. [8] demonstrate that fasting plasma glucose (FPG) significantly increased in subjects with elevated (and untreated) SUA level (> 1 mg/dL). Conversely, it has been reported that SUA level was higher in the pre-diabetic population but lower in people recently diagnosed with diabetes than in prediabetic or normoglycemic persons [9, 10]. The causal association between the phenomena remains unsolved. In addition, despite a strong association between SUA level and various disorders in humans, since UA has the ability to act as a powerful scavenger of free radicals (i.e., antioxidant), elevated SUA has been instead considered as a beneficial phenomenon [11], as it has a compensatory role [e.g., antioxidant in response to increased oxidative stress in conditions such as hypertension, type 2 diabetes, and cardiovascular disease (CVD)] [12]. We have previously demonstrated that SUA is more strongly associated with IFG [13] and MetS [14] in women than in men, and its role as a risk factor may be stronger in women than in men. To our knowledge, there are few studies on the relationship between changes in SUA and long-term glycemic control among community-dwelling persons.

Firstly, this study investigated the relationships between baseline and changes in SUA, and follow-up HbA1c as well as their potential confounding factors. Secondly, we determined whether baseline and changes in SUA are interactively association with follow-up HbA1c. To examine these two issues, we used retrospective data from community-dwelling persons.

## Methods

### Subjects

The present study was a retrospective cohort designed as part of the Nomura study [15]. We have identified and compared people who underwent a similar examination 11 years prior to this examination (in 2002 and 2013). The study population was selected through a community-based annual check-up process from the Nomura health and welfare center in a rural town located in Ehime prefecture, Japan. Participants taking antidiabetic and SUA lowering medication at baseline were excluded. The study complies with the Declaration of Helsinki, and was approved by the ethics committee of Ehime University School of Medicine with written informed consent obtained from each subject (Institutional Review Board: 1402009).

### Evaluation of confounding factors

Information on demographic characteristics and risk factors was collected using the clinical files. Body mass index was calculated by dividing weight (in kilograms) by the square of the height (in meters). We measured blood pressure with an appropriate-sized cuff on the right upper arm of the subjects in the sedentary position using an automatic oscillometric blood pressure recorder while they were seated after having rested for at least 5 min. Information on medical history, present conditions, smoking status, alcohol consumption, and medications (e.g., antihypertensive, lipid-lowering, antidiabetic, and SUA lowering medications) were obtained by interview using a structured questionnaire. Smoking status was defined as the number of cigarette packs per day multiplied by the number of years smoked (pack-year), and the participants were classified into never smokers, past smokers, light smokers (< 20 pack-year) and heavy smokers (≥ 20 pack-year). Daily alcohol consumption was measured using the Japanese liquor unit in which a unit corresponds to 22.9 g of ethanol, and the participants were classified into never drinkers, occasional drinkers (< 1 unit/day), daily light drinkers (1–2 units/day), and daily heavy drinkers (2–3 units/day). Total cholesterol (T-C), triglycerides (TG), high-density lipoprotein cholesterol (HDL-C), FPG, HbA1c, and SUA were measured by the laboratory of the health examination center during an overnight fast of more than 11 h. Plasma samples were immediately frozen and stored at − 30 °C until measurements were taken at the laboratory in our department. Low-density lipoprotein cholesterol (LDL-C) level was calculated by the Friedewald formula [16]. Participants with TG levels ≥ 400 mg/dL were excluded. Estimated glomerular filtration ratio (eGFR) was calculated using CKD-EPI equations modified by a Japanese coefficient: men, Cr ≤ 0.9 mg/dL, 141 × (Cr/0.9)^−0.411^ × 0.993^age^ × 0.813; Cr > 0.9 mg/dL, 141 × (Cr/0.9)^−1.209^ × 0.993^age^ × 0.813; women, Cr ≤ 0.7 mg/dL, 144 × (Cr/0.7)^−0.329^ × 0.993^age^ × 0.813; Cr > 0.7 mg/dL, 144 × (Cr/0.7) ^−1.209^ × 0.993^age^ × 0.813 [17]. Diabetes was defined as a FPG ≥ 126 mg/dL, HbA1c ≥ 6.5%, or use of hypoglycemic medication. Moreover, ischemic stroke, ischemic heart disease, and peripheral vascular disease were defined as CVD.

11-year changes in various factors were calculated by subtracting the baseline values from the 11-year values. A baseline SUA level (women: < 4.0 mg/dL; men: < 5.3 mg/dL) was defined as normouricemia based on the 1st tertile of the baseline SUA (Additional file 1: Figure S1) and a change in SUA (women: < 0.8 mg/dL; men: < 0.4 mg/dL) was defined as a low change based on the 1st and 2nd tertiles.

### Statistical analysis

Data are presented as the mean ± standard deviation (SD) unless otherwise specified, and in the cases of parameters with non-normal distributions (e.g., TG, HbA1c) the data are shown as median (interquartile range) values. In all analyses, parameters with non-normal distributions were used after log-transformation. Statistical analysis was performed using IBM SPSS Statistics Version 20 (Statistical Package for Social Science Japan, Inc., Tokyo, Japan). Correlations between characteristics and HbA1c were determined using Pearson’s correlation. Subjects were divided into four groups based on the tertiles of baseline and changes in SUA within gender (i.e., women: Group 1, baseline SUA < 4.0 mg/dL and changes in SUA < 0.8 mg/dL; Group 2, ≥ 4.0 mg/dL and < 0.8 mg/dL; Group 3, < 4.0 mg/dL and ≥ 0.8 mg/dL; Group 4, ≥ 4.0 mg/dL and ≥ 0.8 mg/dL; men: Group 1, baseline SUA < 5.3 mg/dL and changes in SUA < 0.4 mg/dL; Group 2, ≥ 5.3 mg/dL and < 0.4 mg/dL; Group 3, < 5.3 mg/dL and ≥ 0.4 mg/dL; Group 4, ≥ 5.3 mg/dL and ≥ 0.4 mg/dL), and differences among the groups were analyzed by ANOVA for continuous variables or the Wilcoxon signed rank test for categorical variables. The interactive effect between baseline SUA and changes in SUA on follow-up HbA1c was evaluated using a general linear model. ANCOVA was performed using a general linear model approach to determine the association between confounding factors and follow-up HbA1c. Moreover, to examine the consistency of the observed association between baseline SUA and changes in SUA on follow-up HbA1c, we performed subgroup analyses by age (≥ 50, < 50 years), antihypertensive medication (yes, no), and eGFR (≥ 90, < 90 mL/min/1.73 m^2^). A *p* value < 0.05 was considered significant.

## Results

### Baseline and follow-up characteristics

Baseline and 11-year follow-up characteristics of subjects are illustrated in Table 1. The subjects comprised 393 women aged 71 ± 8 years and 279 men aged 71 ± 10 years. In women, age, alcohol consumption, systolic blood pressure (SBP), presence of antihypertensive medication, HDL-C, presence of lipid-lowering medication, SUA, prevalence of diabetes, and HbA1c were significantly increased after the 11-year follow-up, but BMI, smoking status, diastolic blood pressure (DBP), and eGFR were significantly decreased. There was no inter-group difference regarding prevalence of CVD, TG, and LDL-C. In men, age, alcohol consumption, prevalence of CVD, presence of antihypertensive medication, HDL-C, LDL-C, presence of lipid-lowering medication, prevalence of diabetes, and HbA1c were significantly increased, but BMI, smoking status, DBP, TG and eGFR were significantly decreased.

**Table 1:** Baseline and 11-year follow-up characteristics in women and men

Characteristics N = 393	Baseline	Follow-up	Change	*p*-value*
Women				
Age (years)	59 ± 8	71 ± 8	12 ± 0.5	*< 0.001*
Body mass index (kg/m^2^)	23.2 ± 3.0	22.4 ± 3.2	− 0.8 ± 1.8	*< 0.001*
Smoking status^a^ (%)	58.0/36.4/5.3/0.3	97.7/1.5/0.5/0.3	39.3/− 34.9/− 4.7/0	*< 0.001*
Alcohol consumption^b^ (%)	98.2/0.8/1.0/0	70.0/24.0/3.6/2.3	− 28.2/23.2/2.6/2.3	*< 0.001*
History of CVD, N (%)	2.8	4.6	1.8	0.108
Systolic blood pressure (mmHg)	132 ± 22	137 ± 17	5 ± 19	*< 0.001*
Diastolic blood pressure (mmHg)	78 ± 12	77 ± 9	− 1 ± 11	*0.008*
Antihypertensive medication (%)	16.0	45.0	29.0	*< 0.001*
Triglycerides (mg/dL)	87 (65–114)	87 (65–116)	− 2 ± 49	0.352
HDL cholesterol (mg/dL)	65 ± 16	69 ± 17	4 ± 12	*< 0.001*
LDL cholesterol (mg/dL)	127 ± 31	124 ± 29	− 3 ± 37	0.065
Lipid-lowering medication (%)	6.1	32.6	26.5	*< 0.001*
eGFR (mL/min/1.73 m^2^)	83.1 ± 16.6	71.0 ± 12.4	− 12.1 ± 11.8	*< 0.001*
Serum uric acid (mg/dL)	4.4 ± 1.0	4.8 ± 1.2	0.4 ± 0.9	*< 0.001*
Diabetes (%)	2.3	7.4	5.1	*< 0.001*
HbA1c	4.8 (4.7–5.0)	5.7 (5.5–5.9)	0.8 ± 0.4	*< 0.001*

Characteristics N = 279	Baseline	Follow-up	Change	*p*-value*
Men				
Age (years)	59 ± 10	71 ± 10	12 ± 0.5	*< 0.001*
Body mass index (kg/m^2^)	23.5 ± 2.8	23.0 ± 2.9	− 0.5 ± 1.6	*< 0.001*
Smoking status^a^ (%)	9.7/28.3/35.8/26.2	44.1/39.4/4.7/11.8	34.4/11.1/-31.1/-14.4	*< 0.001*
Alcohol consumption^b^ (%)	45.5/25.1/9.0/20.4	24.0/21.9/18.6/35.5	− 21.5/− 3.2/9.6/15.1	*< 0.001*
History of CVD, N (%)	6.1	10.8	4.7	*0.009*
Systolic blood pressure (mmHg)	135 ± 20	135 ± 17	− 0 ± 20	0.715
Diastolic blood pressure (mmHg)	84 ± 12	79 ± 11	− 4 ± 13	*< 0.001*
Antihypertensive medication, N (%)	18.3	46.6	28.3	*< 0.001*
Triglycerides (mg/dL)	100 (73–139)	89 (67–126)	− 18 ± 79	*< 0.001*
HDL cholesterol (mg/dL)	58 ± 14	60 ± 15	2 ± 12	*0.010*
LDL cholesterol (mg/dL)	109 ± 33	114 ± 30	5 ± 31	*0.008*
Lipid-lowering medication, N (%)	2.2	12.9	10.7	*< 0.001*
eGFR (mL/min/1.73 m^2^)	83.0 ± 16.7	69.7 ± 12.2	− 13.2 ± 12.8	*< 0.001*
Serum uric acid (mg/dL)	5.8 ± 1.3	5.9 ± 1.3	0.1 ± 1.1	0.105
Diabetes, N (%)	5.7	12.5	6.8	*0.001*
HbA1c	4.9 (4.7–5.3)	5.6 (5.4–6.0)	0.7 ± 0.7	*< 0.001*

Change, 11-year follow-up—Baseline

*CVD* cardiovascular disease, *HDL* high-density lipoprotein, *LDL* low-density lipoprotein, *eGFR* estimated glomerular filtration rate, *HbA1c* hemoglobin A1c

^a^Smoking status was defined as the number of cigarette packs per day multiplied by the number of years smoked (pack-year), and the participants were classified into never smokers, past smokers, light smokers (< 20 pack-year) and heavy smokers (≥ 20 pack-year)

^b^Alcohol consumption was measured using the Japanese liquor unit in which a unit corresponds to 22.9 g of ethanol, and the participants were classified into never drinkers, occasional drinkers (< 1 unit/day), daily light drinkers (1–2 unit/day), and daily heavy drinkers (2–3 unit/day). Data presented are mean ± standard deviation. Data for triglycerides and HbA1c were skewed and presented as median (interquartile range) values, and were log-transformed for analysis

* *p*-value: paired t-test for continuous variables or the Wilcoxon signed rank test for categorical variables. Significant values (*p *< 0.05) are presented in italics

### Baseline characteristics of participants categorized by baseline and changes in SUA

The baseline characteristics of the participants categorized by baseline and changes in SUA are illustrated in Table 2. In women, baseline BMI, SBP, DBP, presence of antihypertensive medication, and TG were significantly higher in Group 4 than in the other Groups, but eGFR was significantly lower. In men, baseline BMI, alcohol consumption, prevalence of CVD, TG, and presence of lipid-lowering medication were significantly higher in Group 4 than in Group 1, but eGFR was significantly lower.

**Table 2:** Baseline characteristics of women and men categorized by baseline and changes in serum uric acid

	Group 1	Group 2	Group 3	Group 4	*p*-value*
Baseline serum uric acid	< 4.0 mg/dL	≥ 4.0 mg/dL	< 4.0 mg/dL	≥ 4.0 mg/dL	
Changes in serum uric acid	< 0.8 mg/dL	< 0.8 mg/dL	≥ 0.8 mg/dL	≥ 0.8 mg/dL	
Baseline characteristics N = 393	N = 88	N = 193	N = 49	N = 63	
Women					
Age (years)	59 ± 8	59 ± 7	60 ± 8	60 ± 8	0.714
Body mass index (kg/m^2^)	22.0 ± 2.8	23.6 ± 3.1	22.7 ± 2.7	24.3 ± 2.8	*< 0.001*
Smoking status (%)	64.8/31.8/3.4/0	54.9/36.8/7.8/0.5	65.3/34.7/0/0	52.4/42.9/4.8/0	0.391
Alcohol consumption (%)	98.9/0/1.1/0	98.4/1.0/0.5/0	100/0/0/0	95.2/1.6/3.2/0	0.453
History of CVD, %	4.5	2.1	2.0	3.2	0.683
Systolic blood pressure (mmHg)	126 ± 20	133 ± 21	132 ± 22	138 ± 22	*0.003*
Diastolic blood pressure (mmHg)	76 ± 12	79 ± 11	77 ± 11	82 ± 12	*0.007*
Antihypertensive medication (%)	4.5	16.6	14.3	31.7	*< 0.001*
Triglycerides (mg/dL)	79 (62–97)	91 (68–126)	76 (63–95)	97 (63–124)	*0.024*
HDL cholesterol (mg/dL)	66 ± 14	65 ± 16	66 ± 14	64 ± 17	0.767
LDL cholesterol (mg/dL)	124 ± 30	131 ± 30	119 ± 34	126 ± 31	0.057
Lipid-lowering medication (%)	3.4	6.7	4.1	9.5	0.410
eGFR (mL/min/1.73 m^2^)	91.4 ± 15.5	80.1 ± 15.7	84.9 ± 15.8	79.2 ± 17.5	*< 0.001*
Serum uric acid (mg/dL)	3.4 ± 0.5	5.0 ± 0.8	3.5 ± 0.4	4.7 ± 0.6	*< 0.001*

	Group 1	Group 2	Group 3	Group 4	*p*-value*
Baseline serum uric acid	< 5.3 mg/dL	≥ 5.3 mg/dL	< 5.3 mg/dL	≥ 5.3 mg/dL	
Changes in serum uric acid	< 0.4 mg/dL	< 0.4 mg/dL	≥ 0.4 mg/dL	≥ 0.4 mg/dL	
Baseline characteristics N = 279	N = 46	N = 132	N = 49	N = 52	
Men					
Age (years)	62 ± 9	58 ± 11	64 ± 7	58 ± 9	*< 0.001*
Body mass index (kg/m^2^)	22.6 ± 2.8	23.8 ± 2.7	23.0 ± 2.4	24.1 ± 2.9	*0.011*
Smoking status (%)	13.0/32.6/34.8/19.6	8.3/28.8/37.9/25.0	10.2/34.7/32.7/22.4	9.6/17.3/34.6/38.5	0.488
Alcohol consumption (%)	60.9/15.2/4.3/19.6	48.5/25.8/11.4/14.4	38.8/26.5/10.2/24.5	30.8/30.8/5.8/32.7	*0.044*
History of CVD, %	6.5	2.3	4.1	17.3	*0.002*
Systolic blood pressure (mmHg)	134 ± 21	133 ± 19	138 ± 21	138 ± 19	0.298
Diastolic blood pressure (mmHg)	84 ± 11	83 ± 11	83 ± 12	87 ± 11	0.115
Antihypertensive medication (%)	17.4	16.7	20.4	21.2	0.875
Triglycerides (mg/dL)	89 (75–109)	105 (79–155)	82 (57–107)	111 (74–166)	*0.001*
HDL cholesterol (mg/dL)	62 ± 17	58 ± 14	58 ± 13	57 ± 14	0.234
LDL cholesterol (mg/dL)	107 ± 27	110 ± 34	117 ± 33	102 ± 34	0.165
Lipid-lowering medication (%)	2.2	0.8	0	7.7	*0.020*
eGFR (mL/min/1.73 m^2^)	87.0 ± 14.4	80.2 ± 16.3	86.3 ± 19.1	83.2 ± 16.5	*0.042*
Serum uric acid (mg/dL)	4.3 ± 0.8	6.7 ± 0.9	4.5 ± 0.6	6.2 ± 0.7	*< 0.001*

Data presented are mean ± standard deviation. Data for triglycerides was skewed and was presented as median (interquartile range) values, and was log-transformed for analysis

* *p*-value from ANOVA for continuous variables or from Wilcoxon signed rank test for categorical variables. Significant values (*p *< 0.05) are presented in italics

### Follow-up characteristics of participants categorized by baseline and changes in SUA

Follow-up eGFR, SUA, and prevalence of diabetes of participants categorized by baseline and changes SUA are illustrated in Table 3. In both genders, follow-up SUA were significantly higher in Group 4 than in the other Groups, but eGFR was significantly lower.

**Table 3:** Follow-up characteristics of women and men categorized by baseline and changes in serum uric acid

	Group 1	Group 2	Group 3	Group 4	*P*-value*
Baseline serum uric acid	< 4.0 mg/dL	≥ 4.0 mg/dL	< 4.0 mg/dL	≥ 4.0 mg/dL	
Changes in serum uric acid	< 0.8 mg/dL	< 0.8 mg/dL	≥ 0.8 mg/dL	≥ 0.8 mg/dL	
Follow-up characteristics N = 393	N = 88	N = 193	N = 49	N = 63	
Women					
eGFR	76.3 ± 8.1	71.8 ± 11.0	67.3 ± 13.3	63.9 ± 16.2	*< 0.001*
Serum uric acid (mg/dL)	3.6 ± 0.7	4.8 ± 0.8	5.0 ± 0.7	6.2 ± 1.0	*< 0.001*
Diabetes (%)	3.4	7.3	10.2	11.1	0.274

	Group 1	Group 2	Group 3	Group 4	*p*-value*
Baseline serum uric acid	< 5.3 mg/dL	≥ 5.3 mg/dL	< 5.3 mg/dL	≥ 5.3 mg/dL	
Changes in serum uric acid	< 0.4 mg/dL	< 0.4 mg/dL	≥ 0.4 mg/dL	≥ 0.4 mg/dL	
Follow-up characteristics N = 279	N = 46	N = 132	N = 49	N = 52	
Men					
eGFR	72.2 ± 7.9	70.7 ± 12.1	65.3 ± 13.4	69.1 ± 13.2	*0.022*
Serum uric acid (mg/dL)	4.1 ± 0.8	6.1 ± 0.9	5.7 ± 1.0	7.3 ± 0.9	*< 0.001*
Diabetes, N (%)	10.9	12.1	12.2	15.4	0.913

Data presented are mean ± standard deviation

* *p*-value from ANOVA for continuous variables or from Wilcoxon signed rank test for categorical variables. Significant values (*p *< 0.05) are presented in italics

### Follow-up and changes in HbA1c of participants categorized by baseline and changes in SUA

**Fig. 1:**
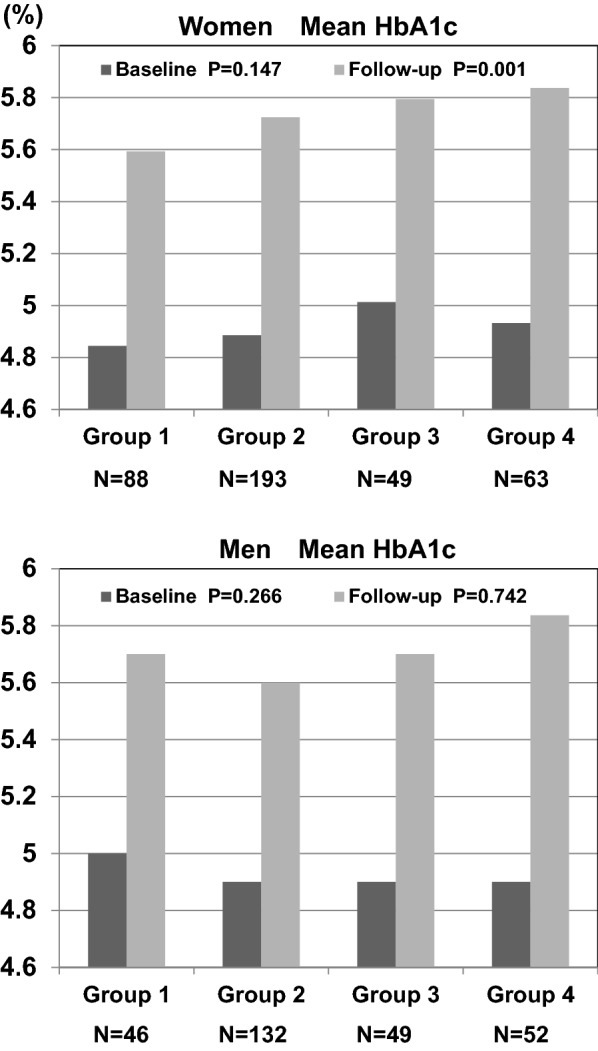
Follow-up and changes in HbA1c of women and men categorized by baseline and changes in serum uric acid. Data for HbA1c were skewed and were log-transformed for analysis. It showed differences among the groups regarding follow-up HbA1c only in women (Group 2, *p *= 0.043; Group 3, *p *= 0.024; Group 4, *p *= 0.001 vs. Group 1 by Bonferroni)

As shown Fig. 1, only in women, there were significant differences among the four groups regarding follow-up HbA1c after 11-year, and follow-up HbA1c was highest in Group 4. However, there were no differences among the four groups regarding baseline HbA1c. In men, there were no differences among the four groups regarding both baseline and follow-up HbA1c.

### Synergistic effect of baseline and changes in SUA on follow-up HbA1c in women

**Fig. 2:**
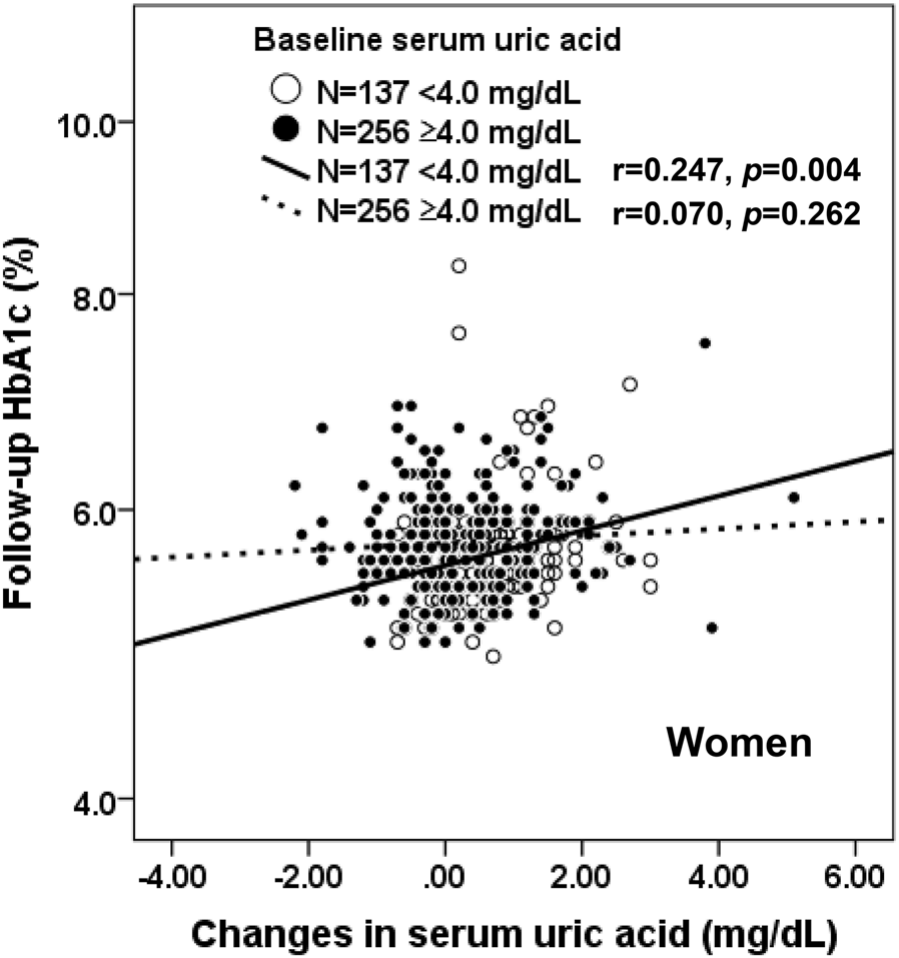
Relationship between changes in serum uric acid and follow-up HbA1c in women categorized by baseline serum uric acid. In the 1st tertile of baseline SUA among women (< 4.0 mg/dL), changes in SUA was significantly correlated with HbA1c over the 11-year follow-up (r = 0.247, *p *= 0.004), but not significantly correlated in the 2nd and 3rd tertiles of baseline SUA (≥ 4.0 mg/dL) (r = 0.070, *p *= 0.262). Analysis of covariance showed that two regression lines in each graph were significantly different (F = 4.698, *p *= 0.031)

In addition to their direct associations, in women we observed a synergistic effect between baseline and changes in SUA. In Fig. 2, when the female participants were divided into two groups according to tertiles of baseline SUA, in the 1st tertile of the baseline SUA group (< 4.0 mg/dL), changes in SUA correlated significantly with follow-up HbA1c after 11 years (r = 0.247, *p *= 0.004), but not significantly in the 2nd and 3rd tertiles of baseline SUA group (≥4.0 mg/dL) (r = 0.070, *p *= 0.262). Analysis of covariance showed that two regression lines in each graph were significantly different (F = 4.698, *p *= 0.031).

### Relationship between baseline characteristics and changes in SUA, and follow-up HbA1c in women

Table 4 shows the relationship between baseline confounding factors including changes in SUA and follow-up HbA1c. BMI, SBP, DBP, presence of antihypertensive medication, TG, LDL-C, HbA1c, SUA and changes in SUA correlated positively while HDL-C correlated negatively with follow-up HbA1c. In addition, we show the statistical significance of the synergistic relationship using a general linear model with all confounding factors and the interaction between baseline and changes in SUA. The interaction between baseline and changes in SUA as well as baseline LDL-C, eGFR, HbA1c, SUA, and changes in SUA was a significant and independent determinant of follow-up HbA1c.

**Table 4:** Relationship between baseline characteristics and changes in serum uric acid, and follow-up HbA1c in women

	Follow up HbA1c	
Characteristics N = 393	r (*p*-value)	F (*p*-value)
Baseline		
Age	0.056 (0.265)	2.449 (0.118)
Body mass index	*0.157 (0.002)*	0.327 (0.568)
Smoking status	− 0.076 (0.132)	0.166 (0.684)
Alcohol consumption	− 0.020 (0.699)	1.544 (0.215)
History of CVD (yes = 1, no = 0)	0.060 (0.237)	1.158 (0.283)
Systolic blood pressure	*0.204 (< 0.001)*	0.981 (0.322)
Diastolic blood pressure	*0.139 (0.006)*	0.850 (0.357)
Antihypertensive medication (yes = 1, no = 0)	*0.200 (< 0.001)*	2.175 (0.141)
Triglycerides	*0.182 (< 0.001)*	1.360 (0.244)
HDL cholesterol	− *0.145 (0.004)*	3.825 (0.051)
LDL cholesterol	*0.227 (< 0.001)*	*13.793 (< 0.001)*
Lipid-lowering medication (yes = 1, no = 0)	0.084 (0.096)	0.325 (0.569)
eGFR (mL/min/1.73 m^2^)	0.030 (0.559)	*10.715 (0.001)*
HbA1c	*0.510 (< 0.001)*	*118.285 (< 0.001)*
Serum uric acid (SUA)	*0.127 (0.012)*	*9.457 (0.002)*
Changes in SUA	*0.101 (0.046)*	*7.757 (0.006)*
Baseline SUA*Changes in SUA	–	*5.391 (0.021)*
R^2^	–	*0.368*

r, Pearson’s correlation coefficient; Data for triglycerides and HbA1c were skewed and were log-transformed for analysis

The net effect of each interaction was estimated using a general linear model. Significant values (*p *< 0.05) are presented in italics

### Multivariate-adjusted changes in and follow-up HbA1c of women categorized by baseline and changes in SUA

Table 5 shows changes in and follow-up HbA1c after adjustment for all confounding factors among the four groups. Multivariate-adjusted changes in and follow-up HbA1c were significantly high in the Group 4.

**Table 5:** Multivariate-adjusted changes in and follow-up HbA1c of women categorized by baseline and changes in serum uric acid

	Group 1	Group 2	Group 3	Group 4	*p*-value*
Baseline serum uric acid	< 4.0 mg/dL	≥ 4.0 mg/dL	< 4.0 mg/dL	≥ 4.0 mg/dL	
Changes in serum uric acid	< 0.80 mg/dL	< 0.80 mg/dL	≥ 0.80 mg/dL	≥ 0.80 mg/dL	
Characteristics N = 393	N = 88	N = 193	N = 49	N = 63	
Multivariate-adjusted changes in HbA1c	0.71 (0.64–0.79)	0.83 (0.78–0.88)	0.86 (0.76–0.96)	0.93 (0.84–1.01)^a^	*0.004*
Multivariate-adjusted follow up HbA1c	5.60 (5.52–5.66)	5.72 (5.67–5.77)^b^	5.74 (5.65–5.84)	5.81 (5.73–5.89)^a^	*0.002*

Data presented are the mean (95% confidence interval) values. Data for triglycerides and HbA1c were skewed and were log-transformed for analysis. Multivariate-adjusted for confounding factors in Table 1

^a^*p *< 0.005; ^b^ *p *< 0.05 vs. Group 1 by Bonferroni. Significant values (*p *< 0.05) are presented in italics

### Relationship between baseline and changes in SUA, and follow-up HbA1c in women within selected subgroups

Next, to control potential confounding factors by baseline age, presence of hypertensive medication, and eGFR, the data were further stratified by age (≥ 50, < 50 years), presence of hypertensive medication, and eGFR (≥ 90, < 90 mL/min/1.73 m^2^) (Table 6). The baseline and changes in SUA, and their interaction were significant and dependently factors only in women aged ≥ 50 years and without hypertensive medication.

**Table 6:** Relationship between baseline and changes in serum uric acid, and follow-up HbA1c in women within selected subgroups

Baseline characteristics N = 393	N	Baseline SUA F (*p*-value)	Change in SUA F (*p*-value)	Interaction Baseline SUA* changes in SUA F (*p*-value)
Age (years)				
≥ 50	355	*10.414 (0.001)*	*5.498 (0.020)*	*3.895 (0.049)*
< 50	38	0.728 (0.402)	0.292 (0.594)	1.089 (0.308)
Antihypertensive medication				
No	330	*8.064 (0.005)*	*6.143 (0.014)*	*4.662 (0.032)*
Yes	63	1.272 (0.265)	1.062 (0.308)	0.348 (0.558)
eGFR				
≥ 90 mL/min/1.73 m^2^	226	1.981 (0.161)	2.567 (0.111)	1.278 (0.260)
< 90 mL/min/1.73 m^2^	235	*6.551 (0.011)*	*4.176 (0.042)*	3.135 (0.078)

Adjusted for the baseline parameters in Table 1. Data for triglycerides and HbA1c were skewed and were log-transformed for analysis

* *p*-interaction was estimated using a general linear model. Significant values (*p *< 0.05) are presented in italics

## Discussion

This study demonstrates the significance of baseline and changes in SUA for 11-year follow-up HbA1c among the screened community-dwelling persons, thus supporting our previous observation in women [13]. We showed that both baseline and changes in SUA as well as baseline LDL-C, eGFR, and HbA1c were significantly and independently associated with follow-up HbA1c after 11-year, and the interaction between baseline and changes in SUA was also a significant and independent determinant of follow-up HbA1c. In addition, multivariate-adjusted changes in HbA1c and follow-up HbA1c were significantly high in participants with a baseline SUA ≥ 4.0 mg/dL and changes in SUA ≥ 0.80 mg/dL. To our knowledge, there are few studies that have indicated these associations of baseline and changes in SUA with long-term follow-up HbA1c among Japanese community-dwelling women.

Several previous studies have reported a possible association between hyperuricemia and the prevalence of IFG. A total of 566 participants (41% men) without diabetes at baseline were evaluated for incident type 2 diabetes 13 years later, and SUA levels independently predicted incidence of type 2 diabetes in participants with IFG [18]. In prospective analyses of 6408 men and 5309 women, the highest quartile of SUA was an independent risk factor for IFG and incident type 2 diabetes only in women [19]. In 1164 women and 971 men without known diabetes, in women SUA was significantly and dependently associated with IFG, newly diagnosed diabetes, and known diabetes, but in men, after multivariable adjustment SUA levels was significantly associated only with IFG [20]. In a prospective study of 13,328 Chinese women and 41,350 Chinese men without diabetes and IFG, there is a higher risk of developing IFG in association with low or high SUA levels for men, but not for women [21]. From a cross-sectional study of 1209 men and 1636 women, we demonstrated that SUA levels were more strongly associated with the different FPG categories in women compared with men. Only in women, the association remained significant for IFG and newly detected diabetes after multivariate adjustment [13]. The causal association between the phenomena remains controversial. The relationship between SUA and incident IFG and type 2 diabetes may be related not only to baseline SUA but also to changes in SUA. Zhang et al. [22] also showed that mean SUA value was strongly and positively related to prediabetes risk, and showed better predictive ability for prediabetes than baseline SUA. In our study, only in women, we showed that both baseline and changes in SUA were significantly and independently associated with follow-up HbA1c, and the interaction between baseline and changes in SUA was also an important determinant of follow-up HbA1c.

The mechanisms by which SUA reflects the risk for glycemic control are not completely understood. Recent studies have reported that elevated UA may also reflect oxidative stress and systemic inflammation [23, 24] and is closely related with the pathogenesis of IFG and type 2 diabetes [23] that impairs insulin receptor substrate 1 and Akt insulin signaling in the liver, skeletal muscle, and adipose tissue [25]. Gersch et al. [26] demonstrated that UA, the most abundant antioxidant in plasma, reacts directly with nitric oxide (NO) in a rapid irreversible reaction resulting in the formation of 6-aminouracil and depletion of NO, which is an important mediator of insulin action, and enhances blood flow and glucose delivery to the skeletal muscle [27]. In addition, UA induces activation of the renin-angiotensin system in human vascular endothelial cells that is followed by oxidative stress [28].

The precise mechanism of gender differences in relationships between SUA and follow-up HbA1c found in this study are still undermined. Several possible pathophysiological mechanisms are proposed as follows; the influence of sex hormones (e.g., estrogen), which can inhibit reabsorption of UA [29], the effect of alcohol consumption [30], which is likely to be more so in men, and the use of antihypertensive medications (e.g., diuretics), which reduce GFR and increase SUA [31]. We however, demonstrated that this result remained significant after adjustment for age, alcohol consumption, and antihypertensive medication. Chou et al. [32] proposed that the SUA level is more important for predicting the degree of insulin resistance in women than in men. In addition the significant sex differences in the relationships of SUA with insulin resistance may explain part of this stronger association between SUA and CVD in women [33]. Although the reasons for these gender differences were still unclear.

The current study also has some limitations. First of all, our retrospective cohort study design does not eliminate potential causal relationships between confounding factors including baseline and changes in SUA, and follow-up HbA1c. Additionally, blood sample (e.g., baseline SUA, changes in SUA, and HbA1c etc.) are based on a single assessment of blood, which may introduce a misclassification bias. Moreover, we could not eliminate possible effects of the underlying diseases, and medication, especially diuretic and lipid-lowering medication use, and renal function on the results.

## Conclusion

In the present study, the possible associations between both baseline and changes in SUA levels, and glycemic control are significant among community-dwelling women. The underlying mechanism behind this relationship is not clear, but seems to be independent of confounding factors. For community-dwelling healthy women, prospective population-based studies are needed to clarify the mechanisms.

## Additional file

**Additional file 1: Figure S1.** Distribution of baseline serum uric acid in women and men

## References

[1] George J, Struthers AD (2009). Role of urate, xanthine oxidase and the effects of allopurinol in vascular oxidative stress. Vasc Health Risk Manag.

[2] Jia Z, Zhang X, Kang S, Wu Y (2013). Serum uric acid levels and incidence of impaired fasting glucose and type 2 diabetes mellitus: a meta-analysis of cohort studies. Diabetes Res Clin Pract.

[3] Kodama S, Saito K, Yachi Y, Asumi M, Sugawara A, Totsuka K, Saito A, Sone H (2009). Association between serum uric acid and development of type 2 diabetes. Diabetes Care.

[4] Lv Q, Meng XF, He FF, Chen S, Su H, Xiong J, Gao P, Tian XJ, Liu JS, Zhu ZH (2013). High serum uric acid and increased risk of type 2 diabetes: a systemic review and meta-analysis of prospective cohort studies. PLoS ONE.

[5] Xu YL, Xu KF, Bai JL, Liu Y, Yu RB, Liu CL, Shen C, Wu XH (2016). Elevation of serum uric acid and incidence of type 2 diabetes: a systematic review and meta-analysis. Chronic Dis Transl Med.

[6] Pfister R, Barnes D, Luben R, Forouhi NG, Bochud M, Khaw KT, Wareham NJ, Langenberg C (2011). No evidence for a causal link between uric acid and type 2 diabetes: a Mendelian randomisation approach. Diabetologia.

[7] Sluijs I, Holmes MV, van der Schouw YT, Beulens JW, Asselbergs FW, Huerta JM, Palmer TM, Arriola L, Balkau B, Barricarte A (2015). A Mendelian randomization study of circulating uric acid and type 2 diabetes. Diabetes.

[8] Cicero AF, Rosticci M, Bove M, Fogacci F, Giovannini M, Urso R, D’Addato S, Borghi C (2017). Serum uric acid change and modification of blood pressure and fasting plasma glucose in an overall healthy population sample: data from the Brisighella heart study. Ann Med.

[9] Hairong N, Zengchang P, Shaojie W, Weiguo G, Lei Z, Jie R, Feng N, Tuomilehto J, Qing Q (2010). Serum uric acid, plasma glucose and diabetes. Diabetes Vasc Dis Res.

[10] Nan H, Dong Y, Gao W, Tuomilehto J, Qiao Q (2007). Diabetes associated with a low serum uric acid level in a general Chinese population. Diabetes Res Clin Pract.

[11] Fabbrini E, Serafini M, Colic Baric I, Hazen SL, Klein S (2014). Effect of plasma uric acid on antioxidant capacity, oxidative stress, and insulin sensitivity in obese subjects. Diabetes.

[12] Glantzounis GK, Tsimoyiannis EC, Kappas AM, Galaris DA (2005). Uric acid and oxidative stress. Curr Pharm Des.

[13] Kawamoto R, Tabara Y, Kohara K, Kusunoki T, Abe M, Miki T (2013). Serum uric acid is more strongly associated with impaired fasting glucose in women than in men from a community-dwelling population. PLoS ONE.

[14] Takayama S, Kawamoto R, Kusunoki T, Abe M, Onji M (2012). Uric acid is an independent risk factor for carotid atherosclerosis in a Japanese elderly population without metabolic syndrome. Cardiovasc Diabetol.

[15] Kawamoto R, Ninomiya D, Kasai Y, Kusunoki T, Ohtsuka N, Kumagi T, Abe M (2016). Serum uric acid is positively associated with handgrip strength among Japanese community-dwelling elderly women. PLoS ONE.

[16] Friedewald WT, Levy RI, Fredrickson DS (1972). Estimation of the concentration of low-density lipoprotein cholesterol in plasma, without use of the preparative ultracentrifuge. Clin Chem.

[17] Horio M, Imai E, Yasuda Y, Watanabe T, Matsuo S (2010). Modification of the CKD epidemiology collaboration (CKD-EPI) equation for Japanese: accuracy and use for population estimates. Am J Kidney Dis.

[18] Kramer CK, von Muhlen D, Jassal SK, Barrett-Connor E (2009). Serum uric acid levels improve prediction of incident type 2 diabetes in individuals with impaired fasting glucose: the Rancho Bernardo Study. Diabetes Care.

[19] Yamada T, Fukatsu M, Suzuki S, Wada T, Joh T (2011). Elevated serum uric acid predicts impaired fasting glucose and type 2 diabetes only among Japanese women undergoing health checkups. Diabetes Metab.

[20] Meisinger C, Doring A, Stockl D, Thorand B, Kowall B, Rathmann W (2012). Uric acid is more strongly associated with impaired glucose regulation in women than in men from the general population: the KORA F4-Study. PLoS ONE.

[21] Liu Y, Jin C, Xing A, Liu X, Chen S, Li D, Feng P, Liu J, Li Z, Wu S (2013). Serum uric acid levels and the risk of impaired fasting glucose: a prospective study in adults of north China. PLoS ONE.

[22] Zhang Q, Bao X, Meng G, Liu L, Wu H, Du H, Shi H, Xia Y, Guo X, Liu X (2016). The predictive value of mean serum uric acid levels for developing prediabetes. Diabetes Res Clin Pract.

[23] Lu W, Xu Y, Shao X, Gao F, Li Y, Hu J, Zuo Z, Shao X, Zhou L, Zhao Y (2015). Uric acid produces an inflammatory response through activation of NF-kappaB in the hypothalamus: implications for the pathogenesis of metabolic disorders. Sci Rep.

[24] Spiga R, Marini MA, Mancuso E, Di Fatta C, Fuoco A, Perticone F, Andreozzi F, Mannino GC, Sesti G (2017). Uric acid is associated with inflammatory biomarkers and induces inflammation via activating the NF-kappaB signaling pathway in HepG2 cells. Arterioscler Thromb Vasc Biol.

[25] McArdle MA, Finucane OM, Connaughton RM, McMorrow AM, Roche HM (2013). Mechanisms of obesity-induced inflammation and insulin resistance: insights into the emerging role of nutritional strategies. Front Endocrinol.

[26] Gersch C, Palii SP, Kim KM, Angerhofer A, Johnson RJ, Henderson GN (2008). Inactivation of nitric oxide by uric acid. Nucleosides Nucleotides Nucleic Acids.

[27] Higaki Y, Hirshman MF, Fujii N, Goodyear LJ (2001). Nitric oxide increases glucose uptake through a mechanism that is distinct from the insulin and contraction pathways in rat skeletal muscle. Diabetes.

[28] Yu MA, Sanchez-Lozada LG, Johnson RJ, Kang DH (2010). Oxidative stress with an activation of the renin-angiotensin system in human vascular endothelial cells as a novel mechanism of uric acid-induced endothelial dysfunction. J Hypertens.

[29] Yahyaoui R, Esteva I, Haro-Mora JJ, Almaraz MC, Morcillo S, Rojo-Martinez G, Martinez J, Gomez-Zumaquero JM, Gonzalez I, Hernando V (2008). Effect of long-term administration of cross-sex hormone therapy on serum and urinary uric acid in transsexual persons. J Clin Endocrinol Metab.

[30] Gordon T, Kannel WB (1983). Drinking and its relation to smoking, BP, blood lipids, and uric acid. The Framingham study. Arch Intern Med.

[31] Ueno S, Hamada T, Taniguchi S, Ohtani N, Miyazaki S, Mizuta E, Ohtahara A, Ogino K, Yoshida A, Kuwabara M (2016). Effect of antihypertensive drugs on uric acid metabolism in patients with hypertension: cross-sectional cohort study. Drug Res.

[32] Chou P, Lin KC, Lin HY, Tsai ST (2001). Gender differences in the relationships of serum uric acid with fasting serum insulin and plasma glucose in patients without diabetes. J Rheumatol.

[33] Kim SY, Guevara JP, Kim KM, Choi HK, Heitjan DF, Albert DA (2010). Hyperuricemia and coronary heart disease: a systematic review and meta-analysis. Arthritis Care Res.

