# Supplementary material for: Baseline and changes in serum uric acid independently predict glucose control among community-dwelling women
Source: Diabetol Metab Syndr. 2018 Jul 13;10:55. doi: 10.1186/s13098-018-0356-z (PMC6043972; doi:10.1186/s13098-018-0356-z)
Supplement: Supplementary file 1 — Additional file 1: Figure S1. Distribution of baseline serum uric acid in women and men [file 13098_2018_356_MOESM1_ESM.docx]

**Women**

**N=393**

**Men**

**N=279**

Figure S1.
